# MEASUREMENT UNCERTAINTIES: REPORT OF AN INTERNATIONAL WORKING GROUP MEETING

**DOI:** 10.6028/jres.092.021

**Published:** 1987-06-01

**Authors:** R. Collé, Pekka Karp

**Affiliations:** National Measurement Laboratory, National Bureau of Standards, Gaithersburg, MD 20899; Measurement Services Office, Technical Inspection Centre, Helsinki, Finland

Experts representing the International Organization for Standardization (ISO), the International Electrotechnical Commission (IEC), the International Organization of Legal Metrology (OIML), and the International Bureau of Weights and Measures (BIPM), met at the International Bureau of Legal Metrology in Paris, October 1–3, 1986, to initiate the development of a guidance document for the treatment and reporting of measurement uncertainties.

The need for such a guidance document has been long felt throughout the international measurement community. In 1980, the BIPM convened a meeting of experts from eleven national measurement laboratories for the purpose of arriving at a uniform and generally acceptable way of assigning uncertainties to measurement data. This BIPM Working Group on the Statement of Uncertainties agreed on a recommendation (Annex 1) which was subsequently adopted by the International Committee of Weights and Measures (CIPM) in October 1981 (Annex 2). The Recommendation consists of five points which provide a general philosophy for reporting uncertainties. In large part, the points are more in the nature of a briefly outlined approach, rather than an explicit specification of algorithms and methods. At the time of the formulation of the Recommendation, it was believed that many further details would have to be addressed and resolved before the recommended approach could be routinely, uniformly, and widely used.

In the past year, the CIPM referred this matter to the ISO since it was felt that this was a more logical international body for trying to achieve agreement and uniformity on the statement of uncertainties within international standardization and metrology organizations. Responsibility was assumed by the ISO Technical Advisory Group (TAG) 4 since it serves as a coordinating mechanism for addressing measurement issues of common interest to the two worldwide standardization bodies, the ISO and the IEC, and the two worldwide metrology organizations, BIPM and OIML. The present working group (ISO TAG 4/WG3) was thus constituted under the terms of reference of ISO TAG 4, and consists of 11 experts nominated by the represented organizations. The Chairman of the working group is Dr. R. Collé of the National Bureau of Standards.

The terms of reference of the working group, as defined by the ISO TAG 4, is:

to develop a document based upon the recommendation of the BIPM Working Group on Uncertainty which provides guidance on the expression of measurement uncertainty for use within standardization, calibration, laboratory accreditation and metrology services. The purpose of such guidance is to promote full information on how uncertainty statements are arrived at and to provide a basis for the international comparisons of measurement results.

At the October meeting, the TAG 4 working group concluded that its task is to produce a document which will be firmly based on the BIPM recommendations of 1980, but will be more specific and usable. The document will be directed towards two primary user groups: national primary standards laboratories and secondary level standards and calibration laboratories. The working group meeting resulted in the completion of a detailed outline for the organization and general contents of a guidance document, as well as a schedule and plan for producing a draft of this document. It is envisaged that a first draft of the document will be discussed at the next TAG 4/WG 3 meeting in May, 1987.

A complete report of the first meeting may be obtained from Mr. David E. Edgerly, Standards Management Program, Building 101, Room A625, National Bureau of Standards, Gaithersburg, MD 20899.

## ANNEX 1 RECOMMENDATION of the Working Group on the Statement of Uncertainties *presented to Comité International des Poids et Mesures*

### Assignment of experimental uncertainties RECOMMENDATION INC-1 (1980)

**1** The uncertainty in the result of a measurement generally consists of several components which may be grouped into two categories according to the way in which their numerical value is estimated:

- those which are evaluated by statistical methods,
- those which are evaluated by other means.

There is not always a simple correspondence between the classification into categories A or B and the previously used classification into “random” and “systematic” uncertainties. The term “systematic uncertainty” can be misleading and should be avoided.

Any detailed report of the uncertainty should consist of a complete list of the components, specifying for each the method used to obtain its numerical value.

**2** The components in category A are characterized by the estimated variances, $s_{i}^{2}$, (or the estimated “standard deviations” *s*_i_) and the number of degrees of freedom, *v*_i_. Where appropriate, the estimated covariances should be given.

**3** The components in category B should be characterized by quantities $u_{j}^{2}$, which may be considered as approximations to the corresponding variances, the existence of which is assumed. The quantities $u_{j}^{2}$ may be treated like variances and the quantities *U_j_* like standard deviations. Where appropriate, the covariances should be treated in a similar way.

**4** The combined uncertainty should be characterized by the numerical value obtained by applying the usual method for the combination of variances. The combined uncertainty and its components should be expressed in the form of “standard deviations.”

**5** If, for particular applications, it is necessary to multiply the combined uncertainty by a factor to obtain an overall uncertainty, the multiplying factor used must always be stated.

## ANNEX 2 RECOMMENDATION CI-1981

The Comité International des Poids et Mesures

Considering

– the need to find an agreed way of expressing measurement uncertainty in metrology,
– the effort that has been devoted to this by many organizations over many years.
– the encouraging progress made in finding an acceptable solution, which has resulted from the discussions of the Working Group on the Expression of Uncertainties which met at BIPM in 1980.

Recognizes

– that the proposals of the Working Group might form the basis of an eventual agreement on the expression of uncertainties,

Recommends

– that the proposals of the Working Group be diffused widely;
– that BIPM attempt to apply the principles therein to international comparisons carried out under its auspices in the years to come:
– that other interested organizations be encouraged to examine and test these proposals and let their comments be known to BIPM;
– that after two or three years BIPM report back on the application of these proposals.

